# Chlamydial Antibiotic Resistance and Treatment Failure in Veterinary and Human Medicine

**DOI:** 10.1007/s40588-016-0028-4

**Published:** 2016-02-03

**Authors:** Nicole Borel, Cory Leonard, Jessica Slade, Robert V. Schoborg

**Affiliations:** Institute of Veterinary Pathology, Department of Pathobiology, Vetsuisse Faculty, University of Zurich, Winterthurerstrasse 268, CH-8057 Zurich, Switzerland; Department of Biomedical Sciences, Center of Excellence for Inflammation, Infectious Disease and Immunity, Box 70579, Quillen College of Medicine, East Tennessee State University, Box 70579, Johnson City, TN 37614-0579 USA

**Keywords:** *Chlamydia trachomatis*, *Chlamydia muridarum*, *Chlamydia suis*, Treatment failure, Tetracycline resistance, Gastrointestinal infection

## Abstract

The *Chlamydiaceae* are widespread pathogens of both humans and animals. *Chlamydia trachomatis* infection causes blinding trachoma and reproductive complications in humans. *Chlamydia pneumoniae* causes human respiratory tract infections and atypical pneumonia. *Chlamydia suis* infection is associated with conjunctivitis, diarrhea, and failure to gain weight in domestic swine. Chlamydial infections in humans and domesticated animals are generally controlled by antibiotic treatment—particularly macrolides (usually azithromycin) and tetracyclines (tetracycline and doxycycline). Tetracycline-containing feed has also been used to limit infections and promote growth in livestock populations, although its use has decreased because of growing concerns about antimicrobial resistance development. Because Sandoz and Rockey published an elegant review of chlamydial anti-microbial resistance in 2010, we will review the following: (i) antibiotic resistance in *C. suis*, (ii) recent evidence for acquired resistance in human chlamydial infections, and (iii) recent non-genetic mechanisms of antibiotic resistance that may contribute to treatment failure.

## Introduction

The *Chlamydiaceae* are Gram-negative, obligate intracellular bacteria with a complex developmental cycle. After the infectious elementary body (EB) enters a host cell, EB-containing endosomes fuse to form a membrane-bound, cytoplasmic inclusion. Within the inclusion, EB develop into larger, non-infectious reticulate bodies (RB). RB use host cell metabolites to grow, and divide. After 30–70 h, RB mature into infectious EB, which are released from the infected host cell. Under adverse environmental conditions, developing *Chlamydiae* may enter a state referred to as persistence or, more recently, as chlamydial stress or the aberrant RB phenotype [1]. Stressed *Chlamydiae* remain viable, but do not develop into EB and are non-infectious. They have a characteristic appearance and are termed aberrant RB/aberrant bodies (AB). Antibiotic exposure is one stressor that can induce this response. For example, penicillin G elicits the AB phenotype for up to 9 months in culture. When penicillin is removed, the *Chlamydiae* resume normal development and produce EB (reviewed in [2, 3]). Exposure to other β-lactam antibiotics, including amoxicillin, induces the AB phenotype in culture [4] and in vivo [5].

*Chlamydiae* cause asymptomatic infection, as well as acute and chronic diseases affecting different tissues, in humans and other animal species. *Chlamydia trachomatis* serovars A-D cause trachoma, the most common infectious form of human blindness. In 2009, there were ≈40 million cases of active trachoma worldwide [6]. *C. trachomatis* serovars D-K and L1-L3 primarily cause human genital tract infections, with ≈105.7 million cases worldwide in 2008 [7]. Manifestations of genital *C. trachomatis* infection range from urethritis and epididymitis in men to cervicitis, infertility, and ectopic pregnancy in women [8]. *C. trachomatis* may be the most costly non-viral sexually transmitted infection, with cases from 2008 alone resulting in a total lifetime direct medical cost of ≈ $516 million in the US [9]. *Chlamydia pneumoniae* causes human respiratory infections and atypical pneumonia. Recent seropositivity studies [10–12] indicate that >50 % of adults have been *C. pneumoniae* exposed, confirming earlier observations [13]. In the USA, azithromycin (AZM) and doxycycline (DOX) are treatments of choice for *C. trachomatis* infections in adults, though erythromycin, levofloxacin, and ofloxacin are alternatives. AZM is recommended for treatment of pregnant women, with amoxicillin and erythromycin as alternatives [14]. Atypical pneumonia is often treated empirically with AZM because it covers multiple organisms, including *C. pneumoniae*. DOX is also a first-line antibiotic for *C. pneumoniae* (reviewed in [15••]).

Chlamydial infections occur in a wide range of animal species, including mammals, birds, fish, marsupials, insects, and amoebae. Pigs are of particular economic importance and can become infected with *Chlamydia suis, Chlamydia pecorum, Chlamydia psittaci,* and *Chlamydia abortus. C. suis* is the major pig pathogen, often occurring in mixed infections with other chlamydial species. Manifestations of *C. suis* infection include respiratory disease, diarrhea, conjunctivitis, and reproductive disorders, while sub-clinical intestinal infections may impair health and cause economic loss (reviewed in [16]). *C. suis* is endemic in pig farms and wild boar populations worldwide. Though zoonotic transmission of *C. suis* from pigs to humans has not yet been demonstrated [16], its DNA has been detected in conjunctival swabs of Nepalese trachoma patients [17] and Belgian slaughterhouse workers [18]. However, the low amount of *C. suis* DNA detected in slaughterhouse employee eye swabs may result from hand-to-eye “contamination” rather than true infection [18, 19]. Though limited information [20–22] is available concerning antibiotic sensitivity/resistance in other veterinary *Chlamydiae*, we will focus on that in *C. suis* because of space constraints.

## Antibiotic Resistance in *C. suis*

The *C. suis* prototype strain S45 was isolated from feces of an asymptomatic pig in Austria in the late 1960s and is tetracycline (TET) sensitive. *C. suis* strains are generally regarded as genetically diverse with variations in virulence, however, genomic data to support this prediction are unavailable. Only one partial draft *C. suis* genome (strain MD56 isolated from a pig with conjunctivitis) has been published [23]. Other *C. suis* strains isolated in the USA, Austria, Germany, and Italy, (except S45) originated from pigs presenting with conjunctivitis, enteritis, respiratory disease, or reproductive failure [16].

Tetracyclines have been used since the 1950s to treat human and animal chlamydial infections, particularly in livestock. In the past, livestock feed has been TET-supplemented to prevent infections and promote growth. Tetracyclines inhibit bacterial protein synthesis by binding to the small ribosomal subunit, have broad-spectrum anti-microbial activity, are inexpensive, and have low toxicity [24, 25]. However, their wide use in pig production has facilitated selection of resistant bacteria, with significant implications for human health. Increasing concerns about this practice led to a ban of the sub-therapeutic application of tetracyclines in Europe in the 1970s [24]. The mechanisms by which bacteria obtain resistance to tetracyclines include efflux pumps, drug-modifying enzymes, target mutation, and the employment of specialized ribosomal protection proteins [25].

Genetically stable TET resistance (Tet^R^) was first described in *C. suis* strains from diseased and apparently healthy pigs in the USA. Eight Tet^R^ strains were isolated from pig farms in Nebraska and Iowa and homotypic Tet^R^ was retained after ten to 15 passages in TET-free medium. Six of these eight strains were also sulfadiazine resistant [26]. Two of these Tet^R^ strains (R19 and R27) grew in culture at up to 4 μg/mL TET, but not at 5 μg/mL. In contrast, *C. suis* S45 was sensitive to 0.25 μg/mL TET. Chlamydial inclusions exposed to increasing TET concentrations contained larger numbers of AB. Upon TET removal, the AB reverted to typical RB and continued normal development. Furthermore, *C. suis* R19 and *C. trachomatis* L2 occupied the same intracellular vacuole when HeLa cells were sequentially infected with both species [27].

The stable *C. suis* Tet^R^ phenotype was later associated with the resistance gene tet*C* [28]. Seven Tet^R^ strains from the USA each contained one of four related, chromosomally-inserted genomic islands. All 7 resistant isolates carried the tet*C* gene, encoding a TET efflux pump, as well as the TET repressor gene tet*R*. The genomic islands also shared high nucleotide sequence identity with other Gram-negative bacterial resistance plasmids. These integrated *C. suis* Tet^R^ genomic island/plasmid-like elements were the first example of antibiotic resistance acquired in an obligate intracellular bacterium through horizontal gene transfer. Three of the four tet*C* islands also carried a novel insertion sequence homologous to the IScs*605* family of insertion sequences of Gram-negative bacteria. All of these genomic islands were inserted at the same position within the chromosome of Tet^R^*C. suis* strains, interrupting a homologue of the invasion gene (inv) from the *Yersiniae* [28].

Given that most *C. suis* strains are TET sensitive, from where did Tet^R^*C. suis* strains acquire these genomic islands? The chlamydial tet*C* islands have >99 % identity with a resistance plasmid (pRAS3.2) from *Aeromonas salmonicida* [28]. The plasmid is integrated into the genomic island IScs*605*, which also encodes the transposase responsible for integration of these genomic islands into the chlamydial chromosome [29]. More recently, the Gram-negative bacterium *Laribacter hongkongenesis*, which is associated with human gastroenteritis and found in freshwater fish, has been viewed as a potential donor for IScs*605*. The *L. hongkongenesis* IScs*605* shared 100 % nucleotide identity to that from *C. suis*. Pig industry feeding practices that rely upon prophylactic TET delivery and use of fish as a feeding source may have facilitated acquisition of DNA from fish bacteria, like *A. salmonicida* or *L. hongkongenesis*, by *C. suis* infecting the pig gastrointestinal (GI) tract (reviewed in [30]). However, the proposed mechanism of acquisition of the Tet^R^ islands by *C. suis* remains speculative.

In vitro experiments have demonstrated horizontal transfer of Tet^R^ from *C. suis* to human clinical strains of *C. trachomatis* following co-culture. In contrast, Tet^R^ transfer from *C. suis* to *Chlamydia caviae*, the guinea pig chlamydial pathogen, was not observed [31]. In a more recent study examining horizontal gene transfer of 16S rRNA in prokaryotic organisms, four strains of *C. trachomatis* were found to have 16S rRNA genes from *C. suis* [32]. These data indicate gene transfer between *C. suis* and *C. trachomatis* occurs in nature and increases concern that antibiotic resistance genes will be transferred into *C. trachomatis*, either from *C. suis* or from other *Chlamydiae*.

After Tet^R^*C. suis* strains were described in the USA, similar strains were reported in Italy [33], Belgium [16], and Switzerland [34]. In Italy, 14 *C. suis* strains isolated from pigs with conjunctival and/or reproductive disorders reared in four different farms carried a tet*C* gene identical to tet*C* from the original US strains [33]. In vitro DOX minimal inhibitory concentration (MIC) and minimal bactericidal concentration (MBC) values ranged from 4–8 μg/ml for 12 of these isolates. Interestingly, two of the 14 tet*C*-positive *C. suis* strains (MS9 and MS14) showed lower MIC and MBC values (0.5 and 1.0 μg/mL, respectively) indicating partial DOX sensitivity [33]. The same 14 *C. suis* isolates were later tested against levofloxacin, DOX, and rifaximin, an antimicrobial that is non-absorbable after oral administration and locally active inside the intestinal tract [35]. Rifaximin showed good in vitro activity against all 14 Tet^R^ strains, with MIC and MBC values from 0.25–1 μg/mL. Levofloxacin MIC and MBC values ranged from 0.5–1 μg/mL, whereas those for DOX ranged from 4–16 μg/mL, except for the MS9 and MS14 strains described above [35].

A recent report by Borel et al. was the first description of Tet^R^*C. suis* isolation from swine with conjunctivitis and diarrhea on a Swiss farm. Ocular and fecal excretion was observed both before and after TET treatment. Though clinical signs disappeared after treatment, *C. suis* was not eliminated and strains harboring the tet*C* gene were positively selected. This rapid selection for Tet^R^*C. suis* strains was surprising and possibly facilitated by close contact between pigs and TET-mediated selective pressure [34]. More recently, Tet^R^*C. suis* strains were identified in sow vaginal/rectal swabs and boar semen obtained from four pig breeder-fattener farms located in Israel, Cyprus, and Belgium reporting reproductive failures. Notably, the Israeli farm used boar semen imported from a German pig insemination center [36]. In another recent study, *C. suis* was detected via real-time PCR in vaginal swabs from Dutch pigs with reproductive failure and in conjunctival swabs of asymptomatic employees from a Belgian pig slaughterhouse. The tet*C* gene was present only in Dutch porcine *C. suis* isolates and not in human isolates from Belgium [19]. In an additional study, three of 15 *C. suis* strains isolated from rectal swabs of Belgian slaughter pigs (from three of ten farms) were tet*C* positive. However, none of the employees’ eyes harbored resistant strains [18]. Thus, whether Tet^R^*C. suis* strains infect humans, thereby potentially facilitating transfer of TET resistance genes to human *Chlamydiae*, remains an open question.

## Antibiotic Resistance and Treatment Failure in Chlamydial Species that Impact Human Health

*C. trachomatis* and *C. pneumoniae* have in vitro sensitivity to a wide range of antibiotic classes, including macrolides, tetracyclines, rifamycins, and quinolones (reviewed in [15••]). In vitro exposure to several β-lactam antibiotics causes *C. trachomatis* RB to convert to the AB phenotype [4]. When cultured in the presence of sub-inhibitory antibiotic concentrations, *C. trachomatis* can become resistant to rifamycins [via mutations in the RNA polymerase β-subunit gene *rpoB*], macrolides [via 23S rRNA gene mutations], and quinolones [via mutations in the DNA gyrase gene *gyrA*]. However, as of 2010, there was no convincing evidence for in vivo development of homotypic resistance in human chlamydial species (reviewed in [30]). More recent studies of chlamydial strains isolated from infected patients after therapy have also failed to identify resistant organisms. Hong et al. compared *C. trachomatis* serovar A/B strains isolated during an Ethopian trachoma control effort. Analysis of seven strains isolated from previously AZM- or TET-treated patients showed no MIC increase for either drug compared to control strains from untreated communities [37]. A larger study confirmed these results, with none of 15 strains isolated post-treatment failure showing DOX or AZM resistance after a Tanzanian mass-treatment program [38•]. Additionally, a study of 24 *C. trachomatis* genital strains collected in Croatia, which has the highest AZM use in Europe, identified no AZM- or DOX-resistant strains [39]. Similar studies conducted from 1994–2000 on *C. pneumoniae* strains isolated from patients with community-acquired pneumonia also revealed no evidence for homotypic resistance (reviewed in [15••]). Thus, despite the observation of acquired Tet^R^ in *C. suis*, human chlamydial species have, fortunately, not yet crossed this Rubicon.

Published data suggest that treatment failure is a significant problem during human chlamydial infections. For example, Golden et al. observed an 8 % failure rate using recommended treatment regimens for genital *C. trachomatis* infection in women who had not reported subsequent sexual activity [40]. More recently, 13.7 % of women experienced treatment failure for *C. trachomatis* genital infection, despite reporting no post-treatment sexual contact and full medication compliance [41]. A recent review suggests treatment failure rates from 5–23 %, depending upon the patient population examined [42]. Although it remains difficult to discriminate from post-treatment reinfection or lack of treatment compliance, most retrospective studies suggest true treatment failure occurs in humans [43]. Since homotypic antibiotic resistance has not yet been documented in *C. trachomatis* or *C. pneumoniae*, investigators are exploring alternative mechanisms including the following: (i) development of heterotypic antibiotic resistance (perhaps due to slower growth in certain environments or entry into a stress response in which the organisms are refractory to antibiotic treatment) and (ii) infection of anatomic sites where *Chlamydiae* are protected from antibiotics. Notably, these mechanisms are not mutually exclusive and could occur in vivo, increasing the difficulty of determining which (if any) contribute to treatment failure.

Heterotypic resistance, in which a subset of individual organisms within a population exhibit reduced antibiotic sensitivity, is one proposed mechanism for treatment failure in humans (reviewed in [42, 44]). Such resistance can be conferred by phenotypic changes in a stressed bacterial population. Bhengraj et al. isolated *C. trachomatis* strains from recurrently infected female patients that had in vitro MIC values for AZM and DOX of up to 8 μg/ml, compared to 0.12–0.25 μg/ml for a sensitive serovar D control strain. In the absence of genetic data, the authors postulate heterotypic rather than homotypic resistance [45]. O’Neill et al. recently published a sensitivity and genomic analysis of two clinical *C. trachomatis* strains (IU824 and IU888) previously reported to be Tet^R^. MIC and titer assays revealed that neither strain exhibited phenotypic Tet^R^ in vitro. Whole genome sequencing did not reveal any known Tet^R^ element, although single nucleotide polymorphisms were observed in the 23S ribosomal RNA (rRNA) gene in both strains. The authors concluded that the observed resistance was heterotypic and unlikely to result from genetic changes [46]. Thus, it seems likely that heterotypic resistance contributes to treatment failures observed in humans.

Several recent cell culture studies have also illuminated mechanisms by which developing *Chlamydiae* might escape antibiotic action. Törmäkangas et al. infected Calu-3 human lung epithelial cells cultured on either semi-permeable inserts (polarized orientation) or plastic dishes (flat, non-polarized cultures) with *C. pneumoniae*. Polarized Calu-3 cultures produced fewer infectious EB than did “flat” cultures, suggesting that *C. pneumoniae* development differs when host cells are grown in the more biologically relevant polarized condition. Notably, the DOX MBC (minimal antibiotic concentration that eliminated EB production) was >33-fold higher in polarized compared to flat Calu-3 cells, indicating that *C. pneumoniae* is less antibiotic sensitive when growing in polarized cells [47]. Oxygen concentration also alters chlamydial antibiotic sensitivity in culture. Although MICs for DOX, AZM, moxifloxacin, and rifampin for *C. trachomatis* L2 are essentially identical under normoxic (20 % O^2^) and hypoxic (2 % O^2^) conditions; MBC assays reveal that DOX and AZM are significantly less effective at reducing EB titer during hypoxia. In contrast, moxifloxacin and rifampin MBCs are similar under hypoxic and normoxic conditions. Hypoxia upregulates expression of the host cellular ATP-binding cassette (ABC) transporter protein MDR-1, which may reduce the anti-chlamydial effect of DOX [48]. Interestingly, when cultures of non-replicating, interferon-γ-stressed *C. trachomatis* are used as drug targets, DOX is more efficient at reducing EB production under hypoxic, compared to normoxic, conditions [49]. Therefore, local differences in O^2^ concentration or infected host cell developmental state could provide protected “pockets” within infected tissues where *Chamydiae* can survive antibiotic exposure.

In culture, chlamydial stress response initiation halts the developmental cycle and the *Chlamydiae* enter a reversible, non-replicating but viable state. Entry into the AB phenotype also increases resistance of (i) *C. pneumoniae* to AZM and ofloxacin [50], (ii) *C. trachomatis* serovar E to AZM [51], and (iii) *C. trachomatis* L2 to DOX [52]. Stressed chlamydiae are more AZM-resistant in vivo as well. Developing *Chlamydiae* within the genital tract of *Chlamydia muridarum*-infected, amoxicillin-treated mice enter the stressed state [5]. Furthermore, the AZM therapy failure rate increases from 9 % in productively infected animals to 22 % in mice infected with amoxicillin-stressed *Chlamydiae* [53•]. Chlamydial forms with morphologic alterations consistent with stress induction have been observed in tissue samples from chlamydia-infected humans [54, 55, 56•], pigs [57] and mice [5, 58]. Notably, nutrient-starvation, as well as interferon and β-lactam antibiotic exposure, all induce chlamydial stress in culture (reviewed in [2, 3]) and occur in vivo. Thus, variation in local conditions may also increase treatment failure in vivo via chlamydial stress-induced heterotypic resistance [42, 44].

Another intriguing possibility is that chlamydiae infecting specific anatomical sites are protected from antibiotics. These tissues then serve as a reservoir from which the genital tract is “reseeded” after treatment cessation. Recent work has provided compelling data indicating the GI tract is one such protected site. Natural GI infection by chlamydial species that infect animals is well documented (reviewed in [59••]). In an extension of previous studies demonstrating long-term chlamydial GI colonization in mice [60, 61], Yeruva et al. demonstrated that oral inoculation with as little as 100 IFU of *C. muridarum* established GI infection in mice for at least 75 days pi [62]. A single oral 80-mg/kg AZM dose eliminated vaginal EB shedding from *C. muridarum* vaginally infected mice but failed to eradicate infectious EB from cecal tissue in orally infected animals. HPLC analyses indicated AZM levels in cervical and cecal tissue were similar, suggesting similar drug penetration in both tissues. Conversely, DOX treatment eliminated both genital shedding from vaginally infected mice and cecal EB production in orally infected mice. The authors concluded that: (i) chlamydiae harbored within the GI tract are more AZM resistant than those in the genital tract and (ii) DOX more effectively eradicates GI chlamydial infection. They also hypothesized that infectious *Chlamydiae* shed from the GI tract after AZM treatment may re-infect the genital tract [63••]. Over 70 % of women with genital chlamydial infection tested positive for rectal infection in the absence of reported anal-receptive intercourse [64, 65], which is consistent with prediction that GI/genital auto-inoculation occurs in humans. Importantly, AZM treatment failure in rectal infections may be as high as 22 % [66]. Both a recent meta-analysis [67••] and a mathematical modeling study [68] support use of DOX rather than AZM for *C. trachomatis* rectal infection treatment. Though the need for additional case controlled studies was stressed, this recommendation was also echoed in a recent review by Hocking et al. [44].

In humans, genital to GI chlamydial transmission seems most likely to occur during oral sex, though if pharyngeal colonization occurs [69], such contact could also promote GI to genital transmission. In contrast, post-treatment GI to genital auto-inoculation in women most likely results from genital contact with EB-containing GI secretions (reviewed in [59••]). If so, post-treatment auto-inoculation should be more frequent in women than men—which is supported by data demonstrating lower treatment failure in genitally infected men than women [40]. In female mice, auto-inoculation is likely mediated by contaminated GI secretions (GI to genital) or grooming (genital to GI). Recent in vivo imaging studies, however, suggest an additional route. Luciferase-expressing *C. muridarum* rapidly colonizes the murine GI tract for up to 100 days after vaginal inoculation. When auto-inoculation is prevented by fitting mice with Elizabethan collars, or *Chlamydiae* are introduced directly into the upper genital tract, GI infection is still observed. These data suggest that *C. muridarum* may spread to the GI tract via a systemic route, though the authors point out that *C. muridarum* and *C. trachomatis* may differ in this respect [70•]. However, it seems prudent to evaluate whether systemic spread of *C. trachomatis* from the genital tract to the GI tract occurs in humans.

## Conclusions

The recent emergence of Tet^R^*C. suis* strains raises concerns that pigs might be a reservoir for chlamydial Tet^R^ determinants. However, detailed assessments of (i) the distribution of Tet^R^ chlamydial strains in wild and domestic pigs, and (ii) herd-related risk factors associated with Tet^R^ acquisition are lacking. The possibility that other animal *Chlamydiae*, such as *C. abortus* or *C. psittaci*, carry TET (or other) resistance determinants is also unexplored. Finally, the largely-environmental chlamydia-like organisms (CLOs), are also emerging pathogens [71•]. Those CLOs tested to date appear to have antibiotic resistance patterns similar to those of the traditional *Chlamydiaceae*, with the exception of *Estrella lausannensis*, which carries a single mutation in the 23S rRNA gene and is AZM-resistant [72]. Unlike *C. trachomatis*, CLOs are also generally fluoroquinolone resistant [73, 74]. Thus, additional studies are needed to determine whether chlamydial veterinary pathogens and environmental CLOs can transfer antibiotic resistance genes to *C. suis*, *C. trachomatis*, or *C. pneumoniae*.

Recent studies suggest treatment failure during human genital infection results from incomplete eradication of simultaneous chronic GI infection, which re-establishes genital tract infection after therapy. Rank and Yeruva proposed a number of interesting mechanisms by which *Chlamydiae* in the GI tract might escape AZM therapy [59••]. However, it is also important to consider the role of (i) varied local GI O^2^ or nutrient concentrations, (ii) infection of GI cells in different developmental states, and/or (iii) induction of AB formation by stressors in the GI environment, the latter of which is consistent with the observation of *C. suis* AB within the GI epithelium of infected pigs [57]. It seems likely that *Chlamydiae* infect multiple host cell types/locations within the GI tract, which vary in physiological status and/or extracellular environment. In certain host cell types or nutrient-rich areas, chlamydial development would progress rapidly—releasing infectious EB. In other host cell types, or more stressful micro-environments, chlamydial development would be slowed or halted by the stress response—which could provide a treatment-resistant reservoir of *Chlamydiae*. Thus, further dissection of the mechanisms by which *Chlamydiae* establish chronic GI infection and evade antibiotic action is warranted. More importantly, though DOX appears more effective than AZM for eradicating GI/rectal chlamydial infection [63••, 67••], more extensive case-controlled studies in humans are a high priority.

Though many advances in understanding chlamydial biology have been made over the last decade, the spectrum of effective anti-chlamydials has remained nearly unchanged (reviewed by [15••]). Sustained AZM therapy has also been associated with increased adverse cardiovascular events in patients [75], raising the possibility that higher or repeated AZM doses to eliminate GI carriage might be contraindicated. Finally, emergence of homotypic antibiotic resistance in human *Chlamydiae* remains a threat. Thus, development of novel anti-chlamydials is a high priority. The dozens of recently identified candidates are beyond the scope of this review but include type III secretion (T3S) inhibitors, chlamydial enzyme inhibitors, and compounds that block essential host cell functions. For example, modified forms of a salicylidene acylhydrazide T3S inhibitor inhibit *C. muridarum, C. pneumoniae*, and *C. trachomatis* serovar D inclusion development and EB production in culture [76]. The peptide deformylase inhibitor GM6001 inhibits *C. trachomatis* L2 inclusion development in culture and reduces vaginal shedding from *C. muridarum-*infected mice by >100-fold [77]. The antiviral compound ST-669 also reduces *C. muridarum*, *C. trachomatis* L2, and *Chlamydia caviae* growth in culture, possibly by a host lipid droplet-dependent mechanism [78]. Interestingly, exposure to JO146, an inhibitor of the chlamydial protease HtrA, significantly reduces *C. trachomatis* EB production when stressed chlamydiae re-enter developmental cycle [79]. JO146 also inhibits growth of *C. trachomatis* clinical strains in culture [80]. Thus, if chlamydial stress induction contributes to treatment failure in vivo, HtrA inhibitors might be used to increase AZM therapy success. Though recent studies indicate that peptidoglycan (PPG) synthesis inhibitors disrupt cell division in both *Chlamydiae* and CLO [81••, 82], caution in targeting PPG synthesis as a potential drug target is warranted due to the observation that β-lactams induce chlamydial stress [4, 83, 84]. Regardless, given the recent rectal/GI infection data, it is particularly important that potential anti-chlamydials be tested for efficacy in animal models of GI and genital infection before proceeding to clinical trials.
